# The Role of NCOA4-Mediated Ferritinophagy in Health and Disease

**DOI:** 10.3390/ph11040114

**Published:** 2018-10-23

**Authors:** Naiara Santana-Codina, Joseph D. Mancias

**Affiliations:** Division of Genomic Stability and DNA Repair, Department of Radiation Oncology, Dana-Farber Cancer Institute, Harvard Institute of Medicine, Room 221, 4 Blackfan Circle, Boston, MA 02215, USA; Naiara_SantanaCodina@dfci.harvard.edu

**Keywords:** NCOA4, ferritinophagy, iron homeostasis, erythropoiesis, ferroptosis, cancer

## Abstract

Nuclear receptor coactivator 4 (NCOA4) is a selective cargo receptor that mediates the autophagic degradation of ferritin (“ferritinophagy”), the cytosolic iron storage complex. NCOA4-mediated ferritinophagy maintains intracellular iron homeostasis by facilitating ferritin iron storage or release according to demand. Ferritinophagy is involved in iron-dependent physiological processes such as erythropoiesis, where NCOA4 mediates ferritin iron release for mitochondrial heme synthesis. Recently, ferritinophagy has been shown to regulate ferroptosis, a newly described form of iron-dependent cell death mediated by excess lipid peroxidation. Dysregulation of iron metabolism and ferroptosis have been described in neurodegeneration, cancer, and infection, but little is known about the role of ferritinophagy in the pathogenesis of these diseases. Here, we will review the biochemical regulation of NCOA4, its contribution to physiological processes and its role in disease. Finally, we will discuss the potential of activating or inhibiting ferritinophagy and ferroptosis for therapeutic purposes.

## 1. Introduction

Iron is required in multiple biological processes [1] including oxygen binding and transport [2], ATP production (as a cofactor in the citric acid cycle and electron transport) [3], and DNA biosynthesis and repair [4,5]. Iron deficiency is the main cause of anemia [6], which causes significant morbidity and mortality worldwide. Likewise, when iron deficiency affects other tissues (skeletal muscle, heart or brain) it can have fatal consequences [4]. Conversely, iron overload can generate Reactive Oxygen Species (ROS) by the Fenton reaction of the Haber-Weiss cycle thereby contributing to the pathophysiology of iron overload in diseases such as hereditary hemochromatosis [7,8]. Thus, cells must establish a delicate balance between iron availability and storage. This is accomplished through the orchestrated action of a network of proteins involved in iron transport/import (transferrin: Tf, transferrin receptor: TfR, DMT1), export (i.e., the iron export transporter, ferroportin: FPN), storage (ferritin: FTH1/FTL), and a host of additional iron homeostasis regulatory circulating and intracellular proteins [3]. Recently, our lab and others identified a role for Nuclear Receptor Coactivator 4 (NCOA4) in regulating iron homeostasis by binding to ferritin and facilitating its autophagic degradation in a process termed “ferritinophagy [9].” This process is itself regulated by intracellular iron levels that promote or inhibit ferritinophagy depending on iron availability and demand [10]. Here, we will review the biochemical regulation of NCOA4, its role in intracellular and systemic iron homeostasis, and its importance in physiological processes (such as erythropoiesis) and in disease. We will discuss the role of ferritinophagy in ferroptosis, a new type of cell death triggered by iron accumulation and lipid peroxidation. Finally, we will explore the therapeutic potential of modulating NCOA4-mediated ferritinophagy.

## 2. NCOA4 Mediates Ferritin Transport to the Lysosome for Degradation

Cellular iron is mainly stored in ferritin, a protein complex composed of 24 subunits of ferritin light and heavy chains (FTL, FTH1). Ferritin can chelate up to 4500 iron atoms in its ferric-Fe(III) form [11]. Iron is incorporated into ferritin via ferritin iron pores as Fe(II), which is further oxidized to Fe(III) by FTH1 inside the ferritin cage, leading to inert deposits of Fe(III) that are unavailable for intracellular use or generation of ROS [12]. For utilization, iron needs to be released from ferritin and reduced again to Fe(II) [13]. While there are studies showing iron can exit ferritin via the ferritin pore, the most likely mechanism of iron release involves proteolytic degradation of the ferritin shell. This degradation process takes place primarily in the lysosome [14] although some minor proteasomal contribution has been observed [15].

Multiple groups reported ferritin degradation as dependent on autophagy, a conserved catabolic cellular process that degrades cellular proteins and damaged organelles via the lysosome as part of a recycling and protective process to ensure cellular homeostasis basally and in response to stress [14,16,17] (for in-depth reviews on the molecular mechanisms of autophagy we point the reader to several excellent reviews on the subject [18,19,20]). Canonical autophagy requires the coordinated action of approximately 30 autophagy-related (Atg) proteins to form double-membrane vesicles (autophagosomes) that capture cellular cargo in both non-selective and selective manners for eventual lysosomal degradation. Autophagy initiation requires activation of the ULK complex followed by production of phosphatidylinositol-3-phosphate (PI3P) by the class III PI3K complex (VPS34, Beclin1, and p150) and membrane contribution from ATG9 vesicles. Pro-MAP1LC3B (‘LC3B’ and related ATG8 family members) is cleaved to LC3B-I by ATG4B and further conjugated to phosphatidylethanolamine by ATG7, ATG3 and the ATG5-ATG12-ATG16L1 complex. Lipidated LC3B-II binds to the growing membrane of autophagosomes and contributes to its elongation and fusion and also acts as a recruitment platform for autophagic molecular machinery [16,21]. Autophagosomes fuse with lysosomes thereby delivering cargo for degradation via lysosomal enzymes. Autophagy was initially described as a survival response to nutrient starvation; however, it is now appreciated that autophagy is active basally in all cells and can be induced by multiple stressors, such as hypoxia or ROS [22,23]. Recently, selective autophagic processes have been reported involving specific receptor proteins that target select cargo for autophagic degradation including mitochondria (mitophagy), endoplasmic reticulum (ER-phagy) or pathogens (xenophagy) [16]. Using autophagy-deficient MEFs (Atg7^−/−^), Asano et al. showed that in iron-depleted cells, autophagy is required for ferritin degradation via the lysosome. Based on their work they hypothesized a selective ferritin autophagic process dependent on an as yet undetermined cargo receptor [17]. We recently identified NCOA4 as the cargo receptor targeting ferritin to the autophagosome in a selective autophagic process (ferritinophagy) [9]. 

We used a combination of autophagosome enrichment, stable isotope labelling by amino acids in cell culture (SILAC), and liquid chromatography–mass spectrometry (LC–MS) to profile proteins enriched in autophagosomes. One of the most robustly and reproducibly enriched autophagosomal proteins was NCOA4 [9]. While NCOA4 was originally identified as a nuclear receptor coactivator (discussed below), we demonstrated that NCOA4 localized to autophagosome and lysosome structures. To understand the role of NCOA4 in autophagy, we performed affinity purification-mass spectroscopy and identified ferritin subunits as interacting partners (FTL, FTH1) [9]. We also identified HERC2 and NEURL4, subunits of an E3 ubiquitin ligase complex, but this interaction was not seen in FTH1 immunoprecipitates, suggesting distinct NCOA4-ferritin and NCOA4-HERC2/NEURL4 complexes [9]. NCOA4 colocalized with ferritin in autophagosomes and lysosomes. Critically, NCOA4 depletion inhibited delivery of ferritin to the lysosome and thereby ferritin degradation. Reduced ferritin turnover led to a significant decrease in bioavailable intracellular iron that altered the intracellular iron homeostasis network of proteins [9]. In parallel, Dowdle et al. also identified NCOA4 as an autophagy receptor for ferritin [24].

**Figure 1:**
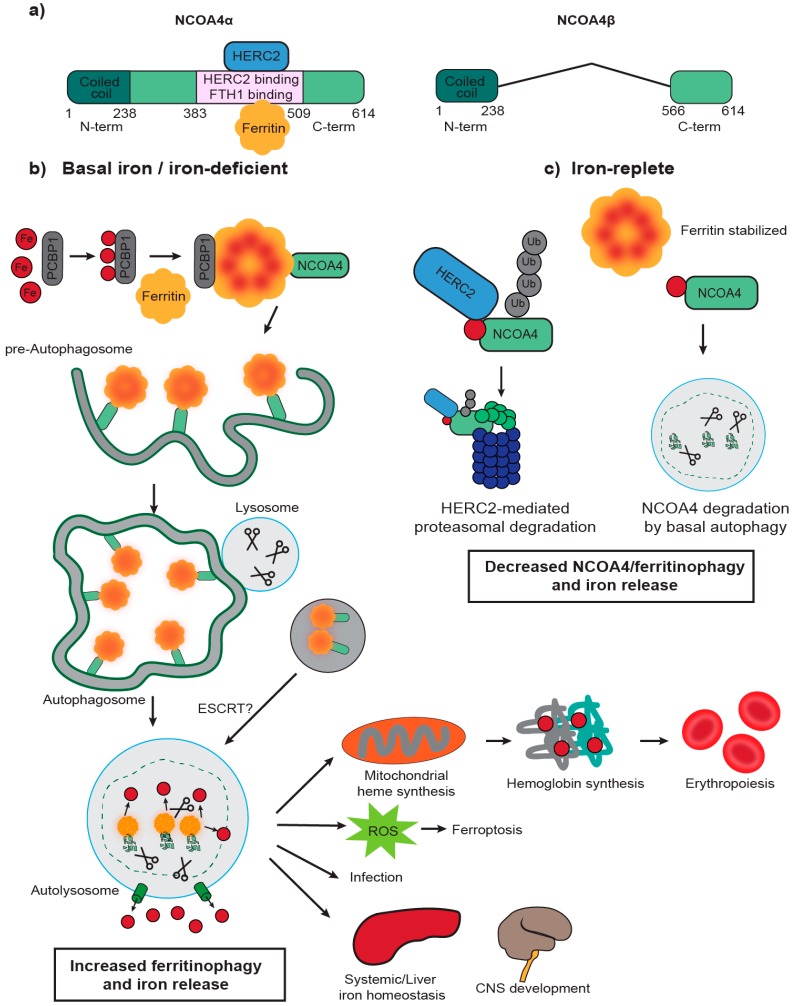
NCOA4-mediated ferritinophagy pathway is regulated by intracellular iron. (**a**) Schematic of NCOA4 transcript variants in humans: NCOA4α and NCOA4β. NCOA4α is a 614 Aa protein (70 kDa) with an N-terminal coiled coil domain and a C-terminal domain that contains a FTH1 and HERC2 binding domain (Aa 383–509). NCOA4β (286 Aa, 35 kDa) shares with NCOA4α the N-terminal domain and a portion of the C-terminal domain. (**b**) Poly rC—binding protein 1 (PCBP1) binds to iron (Fe) and delivers it to ferritin. Fe is stored in ferritin complexes containing ferritin heavy and light chains. NCOA4 binds to ferritin through its C-terminal domain and delivers it to the nascent autophagosome. The mechanisms involved in NCOA4 delivery to the autophagosome are unclear but could involve non-canonical LIR motifs. Fusion of the autophagosome with the lysosome leads to degradation of ferritin and iron release in a process known as “ferritinophagy.” Some studies also suggest alternative pathways for lysosomal delivery of NCOA4-ferritin complexes involving the endosomal pathway (endosomal sorting complex required for transport: ESCRT). Iron is exported to the cytosol where it can be used in several physiological processes that involve iron such as mitochondrial heme synthesis and erythroid differentiation. Increased ferritinophagy flux correlates with reactive oxygen species (ROS) production and a type of cell death known as ferroptosis. Increased ferritinophagy flux has also been observed in certain E. coli urinary tract infections [30]. Finally, iron is also likely required for other physiological processes like liver iron homeostasis and central nervous system (CNS) development, although further research is required to clarify NCOA4’s role in the CNS disease under normal and pathological conditions such as neurodegeneration. (**c**) NCOA4 levels and ferritinophagy are regulated by intracellular iron levels. In iron-replete conditions, HERC2 (an E3 ubiquitin ligase) binds NCOA4 in an iron-dependent manner and mediates its proteasomal degradation. NCOA4 can also be degraded by basal autophagy. Decreased NCOA4 levels lead to stabilization of ferritin by decreased ferritinophagy flux and decreased intracellular levels of iron.

There is limited structural information for NCOA4 except for a N-terminal coiled-coil domain that has been reported as a protein interaction [25] and self-oligomerization domain [26]. Phylogenetic studies show no paralogs for NCOA4, although the ARA70-I family domain (which overlaps with the N-terminal coiled coil domain) is conserved in NCOA4 orthologs through metazoans [27]. NCOA4 has multiple splice variants in rodents and two transcript variants in humans (Figure 1a): NCOA4α (614 Aa, 70 kDa) and the splice isoform NCOA4β (286 Aa, 35 kDa) [28]. The isoforms share the N-terminal coiled-coil domain (Aa 1–238) and a small portion of the C-terminus (Aa 566–614). In vitro assays with recombinant NCOA4 and FTH1 mapped the interaction to the NCOA4 C-terminal domain (Aa 383–509), a domain only present in NCOA4α and not in NCOA4β, and a conserved surface arginine (R23) on FTH1 [10]. An independent study confirmed this interaction and demonstrated that FTH1 can bind up to 24 NCOA4 fragments (Aa 383–522) [29]. Site-directed mutagenesis on NCOA4 identified W497, I489, S492, L494, and L498 as residues important for ferritin binding. Importantly, NCOA4^I489A/W497A^ expression in HCT116 NCOA4 knockout (KO) cells abolished FTH1 binding and ferritin delivery to autophagosomes. Likewise, FTH1^R23A^ mutants prevented an interaction with NCOA4 in cells thereby inhibiting ferritin delivery to the lysosome even under conditions of iron deprivation when ferritinophagy is normally activated. 

Flux through the ferritinophagy pathway is dependent on NCOA4 levels which are tightly regulated by intracellular iron levels (Figure 1b,c) [10]. Under iron-replete cellular conditions, NCOA4 binding to HERC2, an E3 ubiquitin ligase, is increased, leading to proteasomal degradation of NCOA4. Decreased NCOA4 levels inhibit ferritinophagy and increase ferritin iron storage [10]. NCOA4-FTH1 binding appears to be inhibited by high levels of iron, which would similarly decrease ferritinophagy and favor ferritin iron storage [29]. Interestingly, the site for HERC2 binding on NCOA4 (Aa 383–509) overlaps with the site for FTH1 binding raising the possibility that HERC2 and FTH1 binding are mutually exclusive. Future experiments are necessary to determine whether NCOA4 can simultaneously bind HERC2 and FTH1 or whether there is competitive binding. While NCOA4 co-purifies with iron, the mechanism of iron binding and the iron-binding pocket is unclear at this time. Under iron-deficient conditions, presumably when iron occupancy of NCOA4 decreases, HERC2 binding to NCOA4 is decreased, leading to NCOA4 stabilization and induction of ferritin degradation and iron release [10].

The mechanism by which NCOA4-FTH1 reaches the lysosome is not entirely clear. Despite NCOA4 association with multiple ATG8 proteins in in vitro binding assays, no canonical LC3-interaction region (LIR) motif has been identified in NCOA4, although the presence of other non-canonical ATG8-binding motifs may suggest the use of this alternative motif for interaction [31]. While classical autophagy appears to play a prominent role in ferritin delivery to the lysosome, other reports suggest that multiple pathways, including the ubiquitin-proteasome system, can contribute to ferritin degradation. Contribution of each degradation pathway may be different according to cell type or even type of iron deprivation, e.g., one report suggests that ferroportin-mediated iron export promotes ferritin degradation by the proteasome while iron chelation agents induce degradation in the lysosome [32]. Recent studies have suggested an ESCRT complex-mediated endosomal sorting pathway as an alternative, ATG8-independent, lysosomal trafficking pathway for NCOA4 and ferritin. Goodwin et al. report a highly regulated alternative pathway for ferritin degradation involving TAX1BP1, FIP200, ATG9A, VPS34, and components of the ESCRT machinery that is independent of the classical ATG8 macroautophagy pathway [33]. Likewise, the ESCRT-III endosomal pathway has been shown to mediate rapid starvation-induced degradation of autophagy receptors such as NCOA4, p62, NBR1, TAX1BP1, and NDP52 [34]. Further studies are required to explore the mechanism that directs NCOA4 complexes to the lysosome as well as the contribution of different pathways to ferritinophagy. 

While the role of NCOA4 in mediating ferritin degradation and cellular iron homeostasis appears to be a conserved function of NCOA4 across cell types, earlier reports suggested NCOA4 acts as a nuclear receptor coactivator. NCOA4 was originally identified in a yeast-two hybrid screen as a coactivator of the androgen receptor (AR), enhancing its transcriptional activity in prostate cancer [35]. Subsequent studies suggest that NCOA4 also regulates a number of additional nuclear receptors [36,37,38,39,40]. A recent study suggests that NCOA4 can be regulated by thyroid hormone, promoting NCOA4 recruitment to chromatin regions that induce a transcriptional program supporting erythropoiesis [41]. Finally, Bellelli et al. suggest NCOA4 inhibits DNA replication origin activation by binding to the MCM2-7 helicase complex via the NCOA4 N-terminal domain [42]. Further work is required to reconcile these various reported functions of NCOA4.

## 3. NCOA4 Mediates Ferritin Iron Release to Support Erythropoiesis

Given the importance of NCOA4 in intracellular iron homeostasis, several studies have addressed the role of NCOA4 in physiological cellular processes that depend upon iron availability, such as erythropoiesis. Erythropoiesis consists of several differentiation steps between the hematopoietic stem cell progenitor stage and the mature enucleated red blood cell (RBC) that are characterized by gradual degradation of cellular content [43] and production of hemoglobin [12]. Hemoglobinization requires large amounts of iron for heme synthesis with approximately 2 g of iron from the total 3–5 g in the human body incorporated in RBC hemoglobin [3]. Iron is delivered to RBCs by circulating transferrin (Tf) that is internalized via TfR-mediated endocytosis [2]. Next, iron is reduced to its Fe(II) form and exported from the endosome by DMT1. Two different mechanisms by which iron is transported to mitochondria for heme synthesis have been proposed: (1) by direct delivery from the endosome [44] or (2) by export to the cytoplasmic labile iron pool followed by intermediate storage in ferritin, lysosomal ferritin degradation, and delivery to the mitochondria [45,46]. NCOA4 depletion in the K562 human erythroleukemia cell line, an in vitro model of erythroid differentiation, impaired hemoglobinization and differentiation [10], supporting a role for ferritinophagy in mitochondrial iron delivery and heme synthesis. This finding has been confirmed in a murine cellular model of erythropoiesis (G1E-ER4 cells), showing defects in hemoglobinization after NCOA4 depletion albeit without affecting differentiation [47]. These studies support a model whereby iron exported from the endosome enters the cytoplasmic iron pool and is stored in ferritin prior to NCOA4-mediated ferritinophagy and subsequent delivery to the mitochondria for heme synthesis. Based on temporal evaluation of hemoglobinization in their model system, Philpott and collaborators described a model that involves sequential binding of poly rC-binding protein 1 (PCBP1), an iron chaperone, and NCOA4 to ferritin to balance iron storage or use depending on the erythroid differentiation stage [47]. In early erythroid stages, PCBP1 delivers iron to ferritin for storage while low levels of NCOA4 decrease ferritinophagy to further favor iron storage. At the midpoint of erythroid differentiation during the orthochromatic erythroblast stage, a period that correlates with high iron demand and hemoglobin synthesis, PCBP1 and NCOA4 coordinate to increase iron flux through ferritin and ferritinophagy to provide sufficient iron for heme synthesis (Figure 1). Finally, at late stages of erythrocyte development (reticulocyte) as cells lose organelles, iron import is decreased, ferritin levels decrease, and any remaining endosomes may transfer iron directly to mitochondria for ongoing heme synthesis [12,47].

Initial connections between NCOA4 and erythropoiesis on an organismal level were made in zebrafish developmental studies where NCOA4 mRNA expression was found to be upregulated at sites of erythropoiesis [48]. Consistent with this, transcriptional profiling of erythroblasts at sequential stages of differentiation showed that NCOA4 was one of the top upregulated genes in orthochromatic erythroblasts, where hemoglobin synthesis is maximal [49]. NCOA4 depletion in a zebrafish model revealed defects in globin synthesis and hemoglobinization [10]. Using a NCOA4 KO mouse model, Bellelli et al. identified NCOA4-mediated ferritinophagy as critical for iron availability for heme synthesis [50]. NCOA4 KO mice developed a mild hypochromic microcytic anemia together with ferritin and iron accumulation in tissues, indicating inefficient iron mobilization from ferritin. Mice fed an iron-deficient diet developed severe anemia with a compensatory activation of the Erythropoietin (Epo) pathway. Mice fed an iron-enriched diet developed significant accumulation of iron-laden ferritin and excess cytoplasmic iron that precipitated ROS-associated toxicity in the liver and premature death in comparison to controls [50]. This suggests that mice unable to efficiently degrade ferritin may eventually develop an ‘overflow’ of iron into the cytoplasm leading to higher susceptibility to oxidative damage as discussed in greater detail below. NCOA4 appears to be more important at various stages of development as NCOA4 KO mice examined at the immediate postnatal period showed a more severe anemia [41]. This study suggests a differential temporal requirement of NCOA4-mediated ferritinophagy in periods of high iron demand. As the observed anemia is less severe in adult NCOA4 KO mice compared to young mice, this could suggest cell-autonomous or non-autonomous mechanisms that compensate for NCOA4 loss over time. 

NCOA4 KO mice had higher ferritin in serum [50], suggesting that tissues overloaded with iron “leak” ferritin to the blood. This data suggests that there is a mechanism independent of NCOA4-mediated lysosomal delivery and non-classical lysosomal-mediated secretion that promotes ferritin secretion. In agreement with the NCOA4 KO mouse model, NCOA4 depletion in a monocytic cell line did not impair ferritin secretion but in fact increased it, suggesting that NCOA4 is not required for ferritin secretion [51]. Recently, Meyron-Holtz and collaborators demonstrated ferritin secretion via two independent pathways: a non-classical secretory-autophagy pathway and a multivesicular-body-exosome pathway [52].

To date, multiple studies have evaluated the role of NCOA4 in erythropoiesis in vivo but all of them have evaluated it in a systemic KO setting. Despite in vitro evidence in K562 and G1E-ER4 cells suggesting NCOA4 has a cell-autonomous role in erythropoiesis, it remains to be elucidated whether there are non-autonomous contributions of NCOA4 to erythropoiesis. Since NCOA4 knockout is non-fatal in these models, compensatory mechanisms may develop in erythroid cells or other cell types to overcome NCOA4 loss to support hemoglobin synthesis. Identifying these mechanisms will be of interest in the future. Finally, these data support study of NCOA4 in the context of multiple pathological conditions with imbalances in iron homeostasis like hemochromatosis or iron deficiency anemia [53].

## 4. NCOA4-Mediated Ferritinophagy Modulates Ferroptosis 

**Figure 2:**
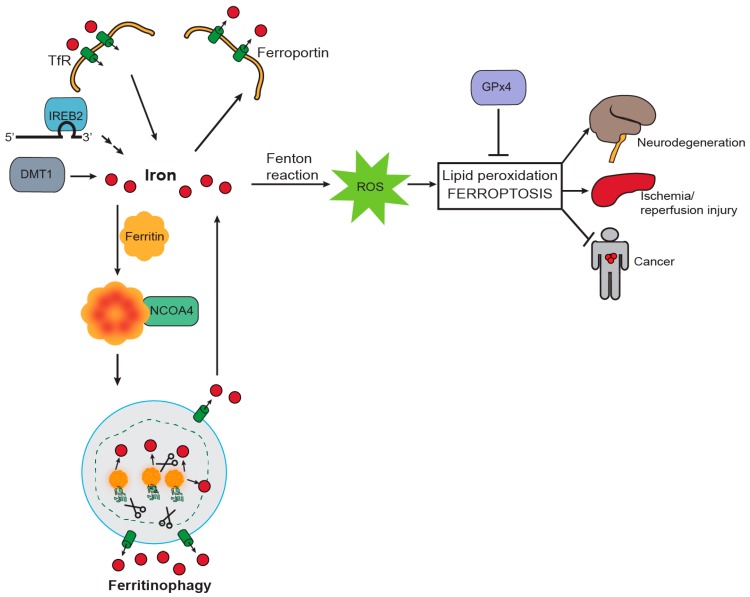
Iron and ferritinophagy are required for ferroptosis induction. Iron release in the cytosol can generate Reactive Oxygen Species (ROS) by the Fenton Reaction. ROS leads to lipid peroxidation and ferroptosis. Iron increase in the cytosol is mediated by increased import (by the transferrin receptor: TfR), decreased export (by ferroportin), increased translation of iron-metabolism related mRNAs via Iron Response Element Binding Protein 2 (IREB2) and increased cytosolic flux by Divalent Metal Transporter 1 (DMT1). Iron can also be stored in ferritin and delivered to the lysosome for NCOA4-mediated iron release from the lysosome. Increased ferritinophagy flux contributes to ferroptosis. Glutathione Peroxidase 4 (GPx4) repairs lipid peroxidation and inhibits ferroptosis. Ferroptosis is a promising therapeutic target. Inhibiting ferroptosis could protect from iron-induced cell death in neurodegeneration and ischemia/reperfusion injury while triggering ferroptosis could be effective in cancer patients.

Ferroptosis is an iron-dependent form of regulated cell death characterized by lipid peroxidation [54,55]. Given the central role of NCOA4-mediated ferritinophagy in regulating intracellular iron levels, ferroptosis sensitivity has recently been shown to be modulated by NCOA4 [56,57]. Ferroptosis is distinct from other identified types of cell death (apoptosis, necrosis or autophagy) at the morphological level, with absence of plasma membrane rupture or blebbing, presence of small mitochondria with reduced cristae, and lack of chromatin condensation [58]. In general, the main ferroptosis-inducing event is lipid peroxidation, which is triggered by inactivation of the lipid repairing phospholipid peroxidase, GPx4 (directly by compounds such as RAS-selective lethal 3 (RSL3) and indirectly by blocking cystine metabolism and glutathione synthesis (erastin, sorafenib and BSO)), or by iron accumulation leading to ROS and lipid peroxidation production (Figure 2). Here, we will focus on how ferroptosis is regulated by alteration in iron homeostasis pathways and specifically NCOA4-mediated ferritinophagy. For in-depth details on the ferroptosis pathway and biochemical regulation, comprehensive reviews are available [54,55,59].

As iron is necessary for the generation of lipid peroxides and thereby initiation of ferroptosis, ferroptosis sensitivity is impacted by compounds that decrease iron availability (iron chelation: deferoxamine (DFO)) and alterations in proteins involved in iron homeostasis that ultimately affect the cellular labile iron pool [58]. For instance, ferroptosis sensitivity is increased with overexpression of TfR (higher import of iron) and decreased expression of FTH1 and FTL (less ability to store iron) [60,61]. As NCOA4 directly regulates ferritin levels and thereby bioavailable levels of iron in the cell, it is not surprising that NCOA4-mediated ferritinophagy has recently been shown to modulate ferroptosis. While early studies were equivocal with respect to the role of autophagy in modulating ferroptosis [49,50], initial reports of NCOA4 depletion showed a decrease in intracellular free iron with a concomitant decrease in oxidative stress [9] accompanied by an increase in glutathione production [56]. Accordingly, NCOA4 deletion inhibited ferroptosis by blocking ferritinophagy and ferritin degradation [56,57]. Conversely, NCOA4 over-expression increased sensitivity to ferroptosis [56]. In fact, treatment with sorafenib, a ferroptosis-inducing drug [54,62], increased NCOA4 expression correlating with its role as mediator of ferroptotic cell death by promoting ferritinophagy and ROS production [63]. While the authors report upregulation of ELAVL1, a transcriptional regulator, as necessary for induction of autophagy/ferritinophagy, it is unclear whether there is a direct relationship between sorafenib, ELAVL1 function, NCOA4 expression levels, and flux through the ferritinophagy pathway [63]. 

Interestingly, Bellelli et al. showed the in vivo relevance of altering ferritinophagy to the ferroptosis pathway. They showed that NCOA4 depletion in mice fed with an iron-rich diet increased expression of GPx and SOD, likely due to overloaded FTH1 leading to iron leakage in tissues followed by upregulation of GPx and SOD as a compensatory mechanism to cope with oxidative stress [50]. Additional studies are required to address the in vivo regulation of autophagy/ferritinophagy as it relates to their contribution to ferroptosis. Also, it remains an open question whether ferroptosis occurs as part of a normal physiological process, perhaps during development.

## 5. Ferritinophagy and Ferroptosis in Disease 

Ferroptosis has been linked to several pathological conditions including neurodegenerative disease, ischemia/reperfusion injury and cancer. As such, there is significant interest in identifying pharmacologic means of blocking (neurodegenerative disease, reperfusion injury) or triggering (cancer) ferroptosis for therapeutic purposes. Given NCOA4 can modulate ferroptosis sensitivity, understanding whether NCOA4 has a role in the pathophysiology of these various diseases is critical.

### 5.1. Neurodegenerative Disease

Neurodegenerative diseases (ND) have historically been associated with inappropriate iron accumulation and oxidative stress. More recently, ferroptosis has been shown to play a part in neurodegeneration pathophysiology [64]. In Parkinson’s Disease (PD), increased iron accumulation and ROS lead to dopamine oxidation, contributing to an oxidative state that promotes dopaminergic neuronal cell death [65]. Elevated DMT1 expression [66] and activating mutations in Tf both lead to increased cytosolic iron and are correlated with worse outcomes for PD patients. Conversely, mutations in TfR2 that decrease cellular iron import correlate with better disease outcomes [67]. Similar observations have been made in Alzheimer’s Disease (AD) [55], where iron is found to co-aggregate with pathogenic amyloid deposits [68]. Indeed, immunofluorescence of brains from patients with AD showed decreased expression of ferroportin, the iron export transporter [69]. Direct evidence that ferroptosis can modulate neuronal cell death initially came from in vitro rat organotypic hippocampal slice cultures treated with glutamate to mimic the pathophysiology of stroke and ND [58]. Glutamate induced ferroptosis that could be prevented by iron chelators and ferrostatin [58]. Studies with genetic [70] or pharmacological inhibitors of ferroptosis [71,72] in a variety of in vitro models and multiple ND disorders (PD, AD, Huntington’s) subsequently confirmed that inhibition of ferroptosis is a potential strategy to prevent neuronal cell death.

To date there is no direct evidence linking ferritinophagy, ferroptosis, and ND. While elevated ferritinophagy sensitizes cells to ferroptosis, the data connecting autophagy and ND instead suggest that decreases in global autophagy activity are correlated with development of ND. Indeed, defective autophagy can cause ND in genetic mouse models of autophagy loss [73,74]. Mutation in *WDR45*, leading to decreased autophagy, has been identified in a number of patients with Neurodegeneration with Brain Iron Accumulation (NBIA) [75]. In general, as autophagy levels decrease with age [76], iron levels increase [77]. As defective autophagy is associated with ND, this suggests a more complex relationship of ferritinophagy activity to ND. Interestingly, in long term NCOA4 KO, mice have increased serum iron and were more sensitive to iron overload [50]. This could suggest that long-term ablation of ferritinophagy in vivo leads to an iron overload phenotype, albeit with decreased utilization of iron for erythropoiesis leading to anemia. Additional studies are necessary to clarify the expression, regulation and function of NCOA4 in the central nervous system (CNS). Given the possible role of ferritinophagy in mediating neuronal cell death, further studies will clarify NCOA4’s role in ferroptosis in the CNS and establish the potential of ferroptosis inhibition in ND, either by iron chelation or by modulating ferritinophagy flux.

### 5.2. Cancer

Many cancers accumulate large amounts of iron to support increased proliferation. This is accomplished in a cell-autonomous manner by increasing iron import or decreasing export [78,79,80,81,82] and in a non-autonomous manner by increasing iron supply from other cell types like macrophages [83,84]. Initial data suggests NCOA4 may be relevant to tumorigenesis and that the intersection of the ferritinophagy and ferroptosis pathways may represent a therapeutic avenue. A positive correlation between NCOA4 mRNA and NCOA4α protein levels and transformation has been described in ovarian carcinoma [85,86]. Here, overexpression of several oncogenes (MYC, H-Ras, p53 inactivation) in normal endometriotic cells to induce transformation led to an upregulation of NCOA4α and NCOA4β expression. Interestingly, NCOA4α knockdown in transformed cells decreased survival whereas NCOA4β overexpression decreased colony formation. On the other hand, in studies of prostate cancer, NCOA4α acts as a tumor suppressor while NCOA4β expression correlated with proliferation and invasion [87]. Similar roles were identified for the two isoforms in MCF7 breast cancer cells [88]. Whether NCOA4 functions differently depending on the cancer context and understanding whether the ferritinophagy specific function of NCOA4 drives tumor effects are critical questions for future study.

While robust evidence for the role of ferritinophagy in tumorigenesis is lacking, there is a significant evidence for the importance of autophagy in cancer with recent links between ferroptosis sensitivity and tumor autophagy dependence. The role of autophagy in cancer is exceedingly complex with autophagy acting in a tumor suppressive or tumor promoting manner depending on the tumor context (reviewed in detail [21]). Autophagy is activated in a variety of cancers [21,89,90,91,92,93] to support metabolism, redox homeostasis and probably iron turnover. In particular, many Kras-driven tumors, such as pancreatic cancer, depend on high autophagic flux [89] and have significant iron accumulation due to elevated TfR expression [94]. Notably, pancreatic cancer cell lines have increased NCOA4 expression and a corresponding high flux through the ferritinophagy pathway [9]. These baseline characteristics would suggest a high sensitivity to ferroptosis. In fact, ferroptosis inducers erastin and RSL3/5 were originally discovered as part of a synthetic lethality screen to determine synergies with the Ras oncogene [60,95]. More direct evidence for the role of NCOA4 in modulating ferroptotic cell death in pancreatic cancer cells came from Yang et al. who showed that artesunate-mediated ferroptotic cell death is attenuated by NCOA4 depletion [96]. In general, autophagy (and therefore ferritinophagy) has been shown to be a positive regulator of ferroptosis [56,57]; therefore, triggering ferroptosis in cancers with high autophagy levels might reveal a potential vulnerability. One possible future therapeutic strategy would be to increase iron flux through the ferritinophagy pathway, leading to increased labile iron and ROS, thereby sensitizing cancer cells to ferroptosis-inducing agents.

### 5.3. Infectious Disease

Ferritinophagy is also involved in modulating susceptibility to infectious diseases. Uropathogenic *Escherichia coli* survive by forming reservoirs within urothelial cell autophagosomes. These bacteria traffic with ferritin-bound iron to the autophagosome to support their proliferation. The authors show that ferritin trafficking is NCOA4-dependent and that NCOA4 depletion reduces bacterial load [30]. Autophagy inhibitors and iron chelators were able to reduce bacterial burden and host cell death, suggesting a therapeutic potential to modulating NCOA4-dependent ferritinophagy in certain bacterial infections. Human cytomegalovirus (HCMV) protein pUL38 blocks the function of USP24, a deubiquitinase, to prevent an iron-dependent, endoplasmic reticulum (ER)-stress induced premature cell death [97]. USP24 deubiquitinase activity stabilizes NCOA4 protein thereby promoting ferritinophagy, which in turn increases cellular iron levels promoting iron-dependent ER stress-induced cell death. Therefore HCMV protein pUL38, via inhibition of USP24, decreases ferritinophagy in order to protect HCMV-infected cells from a premature cell death [97]. These studies suggest that modulating NCOA4-mediated ferritinophagy levels may be an effective strategy to inhibit certain bacterial or viral infections.

## 6. Conclusions and Future Directions

NCOA4-mediated ferritinophagy is integral to iron homeostasis in normal and pathological conditions. Significant progress has been made identifying the molecular mechanisms that regulate NCOA4 activity. However, a number of outstanding questions regarding the biochemical regulation of NCOA4 remain. First, how NCOA4 is regulated on a transcriptional and post-transcriptional level has yet to be determined. Importantly, NCOA4 mRNA does not appear to have a 5′ or 3′ Iron Regulatory Element (IRE) that would engage the iron responsive IREB1/2 system of post-transcriptional regulation. NCOA4-FTH1 and NCOA4-HERC2 binding appears to be regulated by iron levels; however, the mode of NCOA4 iron binding as well as the structural requirements for FTH1 and HERC2 binding are unclear. Further, where and when NCOA4 first interacts with FTH1 or HERC2 in the cell is unclear. The precise mechanism of NCOA4-FTH1 delivery to lysosomes is unclear and there may be multiple routes including a canonical autophagic route and/or an ESCRT complex dependent endosomal-lysosomal transport pathway [33,34]. NCOA4 has been reported to localize to and function in the nucleus as a nuclear receptor coactivator and as a regulator at DNA replication origins. How cytoplasmic versus nuclear NCOA4 localization is determined is an unanswered question, especially given NCOA4 lacks a canonical nuclear localization sequence. Interestingly, the two NCOA4 isoforms, NCOA4α and NCOA4β, differ in that NCOA4β does not contain the C-terminal elements for binding FTH1 and HERC2; however, their relative functions and expression levels in cells is undetermined at this time.

The role of NCOA4-mediated ferritinophagy in physiological processes has so far been assessed mainly in the context of erythropoiesis in zebrafish and murine models of systemic NCOA4 depletion. However, these studies have been unable to identify if the impact on erythropoiesis is due to cell-autonomous effects on iron metabolism in erythrocytes or due to impairment of whole body iron availability. Further studies are required to address this question using conditional knockout mouse models that interrogate NCOA4 in red blood cells or in other cell types that contribute to erythropoiesis. Furthermore, results from Gao et al. suggest there might be a temporal variation in NCOA4 dependency [41], with a higher reliance at early developmental stages. Defining the importance of NCOA4 at different developmental stages as well as comparing acute vs. long-term KO will be key to understanding the mechanisms triggered in erythrocytes or other cell types to compensate for NCOA4 loss. In addition, understanding the relative importance of NCOA4-mediated ferritinophagy in other organs and cell types, especially those highly involved in systemic iron homeostasis, such as the liver and the macrophage system, is critical and will depend on conditional NCOA4 KO mouse models.

The role of ferritinophagy in disease is an underexplored area. Based on the importance of NCOA4 in erythropoiesis and systemic iron homeostasis, future studies of NCOA4 function in the context of anemia and hemochromatosis and whether inhibition or upregulation of ferritinophagy can impact these and other disease processes where iron homeostasis is dysregulated will be informative. The recent discovery that ferritinophagy can modulate sensitivity to ferroptosis, an iron-dependent form of cell death linked to neurodegeneration, cancer and ischemia/reperfusion injury, suggests ferritinophagy may be similarly important in these diseases. Understanding how ferritinophagy contributes to ferroptosis sensitivity in each pathological setting in vivo will be key to designing therapeutic interventions that could trigger or block ferroptosis. The recent discovery of NCOA4 has opened a new area of biology with connections to a wide range of physiologic and pathophysiological processes. Future studies will clarify the above described unanswered biochemical and in vivo functional questions fundamental to our understanding of NCOA4 biology.

## Abbreviations

**NCOA4**	Nuclear Receptor Co-Activator 4
**ATP**	Adenosine Triphosphate
**DNA**	Deoxyribonucleic acid
**ROS**	Reactive Oxygen Species
**TfR**	Transferrin Receptor
**DMT1**	Divalent Metal Transporter 1
**FPN**	Ferroportin
**FTH1**	Ferritin Heavy Chain 1
**FTL**	Ferritin Light chain
**Fe**	Iron
**PI3P**	Phosphatidylinositol-3-phosphate
**MEFs**	Mouse Embryonic Fibroblasts
**Atg7**	Autophagy-related protein 7
**SILAC**	Stable Isotope Labeling by Amino-acids in Cell culture
**LC-MS**	Liquid Chromatography-Mass Spectrometry
**HERC2**	HECT And RLD Domain Containing E3 Ubiquitin Protein Ligase 2
**NEURL4**	Neuralized E3 Ubiquitin Protein Ligase 4
**PCBP1**	Poly rC–binding protein 1
**LIR motif**	LC3-interaction region
**ESCRT**	Endosomal Sorting Complex Required for Transport
**CNS**	Central Nervous System
**AR**	Androgen Receptor
**RBC**	Red Blood Cell
**GPx4**	Glutathione Peroxidase 4
**RSL3**	RAS-selective lethal 3
**BSO**	Buthionine Sulfoximine
**DFO**	Deferoxamine
**SOD**	Superoxide Dismutase
**IREB2**	Iron Response Element Binding Protein 2
**ND**	Neurodegenerative Disease
**PD**	Parkinson’s Disease
**AD**	Alzheimer’s Disease
**NBIA**	Neurodegeneration with Brain Iron Accumulation
**HCMV**	Human Cytomegalovirus
